# HIV Research with Men who Have Sex with Men (MSM): Advantages and Challenges of Different Methods for Most Appropriately Targeting a Key Population

**DOI:** 10.3934/publichealth.2017.3.221

**Published:** 2017-05-17

**Authors:** Ana Gama, Maria O. Martins, Sónia Dias

**Affiliations:** Global Health and Tropical Medicine, GHTM, Instituto de Higiene e Medicina Tropical, IHMT, Universidade Nova de Lisboa, UNL, Lisboa, Portugal

**Keywords:** HIV, Men who have sex with men, surveillance, research, sampling methods

## Abstract

The difficulty in accessing hard-to-reach populations as men who have sex with men presents a dilemma for HIV surveillance as their omission from surveillance systems leaves significant gaps in our understanding of HIV/AIDS epidemics. Several methods for recruiting difficult-to-access populations and collecting data on trends of HIV prevalence and behavioural factors for surveillance and research purposes have emerged. This paper aims to critically review different sampling approaches, from chain-referral and venue-based to respondent-driven, time-location and internet sampling methods, focusing on its main advantages and challenges for conducting HIV research among key populations, such as men who have sex with men. The benefits of using these approaches to recruit participants must be weighed against privacy concerns inherent in any social situation or health condition. Nevertheless, the methods discussed in this paper represent some of the best efforts to effectively reach most-at-risk subgroups of men who have sex with men, contributing to obtain unbiased trends of HIV prevalence and HIV-related risk behaviours among this population group.

## 1. Introduction

### 1.1. HIV Epidemic Trends and Burden among Men who Have Sex with Men (MSM)

The Human Immunodeficiency Virus (HIV) infection continues to be a concern in global public health. Surveillance data published by the European Centre for Disease Prevention and Control and the World Health Organization (WHO) Regional Office for Europe indicate that, in 50 of 53 countries in the WHO European Region, 153,407 new HIV diagnoses were reported in 2015, corresponding to a rate of 17.6 per 100,000 populations in this region [1].

The emergence of Acquired Immunodeficiency Syndrome (AIDS) as a global threat has served to highlight the diversity of lifestyles and complexity of socio-cultural subgroups which need to be considered in health policy [2]. In low-level and concentrated epidemics, key populations (which are at increased risk for acquiring and transmitting HIV and other infections due to specific higher-risk behaviours, in combination with interpersonal, socio-political, and cultural contexts) include injecting drug users, female sex workers, and men who have sex with men (MSM) [3],[4].

Against the background of low or declining HIV prevalence in the general population, MSM continue to be disproportionately affected by HIV infection [5],[6]. In recent years, there has been increased concern about newly identified epidemics of HIV infection among MSM in Asia, Africa, and Latin America [7],[8] and the resurgence in HIV infection among MSM in the Western world [9]–[11]. Recent data estimates that 25.6% of new HIV diagnoses reported in the WHO European Region in 2015 were attributed to sex between men [1]. The number of HIV cases among MSM in Europe increased 27% between 2004 and 2009, more than the HIV cases acquired through heterosexual contact or among injecting drug users [12].

Besides biological vulnerability of MSM to HIV infection (unprotected anal sex represents high risk of transmission) [13], the escalation of HIV/sexually transmitted infections (STI) in MSM has been largely attributed to a steady increase in high risk sexual behaviours, such as unprotected sex, and multiple and concurrent sexual partners [14],[15].

### 1.2. HIV Surveillance in MSM: a Crucial yet Challenging Endeavour

The main function of HIV/AIDS surveillance is to provide an understanding of local epidemics, including the source of new infections over time and the behavioural and biological factors driving HIV transmission in order to provide a basis for designing and evaluating appropriate interventions [16].

An essential attribute of public health surveillance is data should be representative (and thus generalizable) of the population under surveillance [17]. In contexts where a disease is highly prevalent in the general population and a large proportion of the population comes into contact with health services, routine reporting by healthcare-providing institutions serves as a surveillance mechanism [16],[18]. In most developing countries, the main sources of routinely available HIV surveillance data have been pregnant women seeking antenatal care and sexually transmitted disease patients [18]. When there is a lack of facility-based data, general population surveys can provide adequate HIV surveillance data, although at a higher cost. Survey-based public health surveillance of health status and related behaviours in general population has been common practice [16]. With regard to HIV/AIDS, population-based surveys have provided much of the general population behavioural surveillance data available in developing countries [16],[18].

In scientifically rigorous endeavours, the preferred approach for surveillance is probability sampling in which sample elements are chosen randomly from a known sample frame [16],[19]. Such conventional surveillance approach traditionally targets the general and most easily accessible populations, but often it only captures a small fraction of the population of some subgroups [16],[19]. In order to be effective, it is crucial that surveillance efforts focus on the segments of populations which play an important role in HIV transmission [16]. The behaviours and HIV status of those covered and missed by conventional surveillance efforts can differ quite substantially. Hence, there is great hazard of surveillance data failing to capture significant pockets of infection that can lead to a more generalized spread of HIV if not contained [16]. Accurately measuring the prevalence of HIV and behaviours over time in key populations, as MSM, is essential to planning and implementing cost-effective, targeted prevention programs [20]–[24].

### 1.3. Sampling of Key and Difficult-to-Access Populations as MSM

A primary challenge for surveillance of key populations is obtaining “representative” samples [16],[25]. In fact, there is no list or sampling frame for these subgroups, which makes it unfeasible to create a useful sampling frame and use traditional probability-based sampling methods [2],[16]. Also, key populations as MSM often represent a small proportion of the general population, therefore obtaining statistically reliable data for such subpopulations through household surveys would require prohibitively large sample sizes [16],[19],[26]. Lastly, when the risk-taking behaviours which justify the inclusion of most-at-risk subgroups in HIV surveillance are considered deviant or illegal within society and are stigmatized, conventional household surveys are unlikely to produce accurate surveillance data [16]. This is the case for MSM. Since the behaviours in which these subpopulations engage are frequently stigmatized, they are generally reluctant to participate in research efforts to measure their infection status and risk behaviours, and subsequently jeopardize revealing personal and sensitive information to others [16],[20],[26],[27].

Therefore, the difficulty in accessing such hard-to-reach populations presents a dilemma for HIV surveillance, as their omission from surveillance systems leaves important gaps in our knowledge and understanding of the HIV/AIDS epidemics [16]. The importance of including these populations has highlighted the need for alternative sampling strategies which are both feasible and capable of producing realistic estimates, with minimal levels of bias for population subgroups which are not efficiently “captured” using conventional surveillance data collection strategies [16],[19]. Special attention has been drawn to the development of sampling methods which provide valid estimates of infection rates, behaviours, and contextual factors among their members for meaningful surveillance. In particular, the use of non-random sampling techniques has been increasingly recognized [2].

Over the past decades, several methods for recruiting difficult-to-access populations for surveillance and research purposes have emerged [28]–[32]. This paper aims to critically review different sampling approaches with a focus on its main advantages and challenges for conducting HIV research among key populations as MSM.

## 2. Sampling Approaches for Conducting HIV Research among Key Populations as MSM

As broadly acknowledged, implementing a simple random sampling approach, though valuable from a statistical perspective, in the context of most vulnerable populations can be extremely expensive, inconvenient or impossible [33]. Alternatively, non-probabilistic methods have been frequently used to reach most-at-risk populations [33].

### 2.1. Chain-referral and Venue-based Sampling Methods

Many HIV surveillance studies conducted in the last decades have relied on non-probability sampling methods, such as chain-referral sampling to recruit members of the target group. These methods work on the assumption that peers are better able to recruit members of a hidden population than researchers [16],[30].

#### 2.1.1. Snowball Sampling

One of the most commonly used methods is Snowball sampling, which has been considered particularly effective in locating members of special populations when the focus of the study is on a sensitive issue [2],[16],[31]. Snowball sampling entails identifying an initial number of subgroup members from whom the desired data are gathered, and who then serve as “seeds” to help identify other subgroup members to be included in the sample, according to the inclusion criteria defined by the researchers. These individuals, in turn, are asked to provide information on other subgroup members, and the process continues until either a target sample size is reached or the sample becomes “saturated” (i.e. new sample subgroup members fail to provide information that differs from that obtained from members interviewed previously) [16]. The snowball sampling can be placed within a set of link-tracing or chain referral methodologies, the basic assumption being the existence of some kind of “linkage” with other people in the sample population [2],[34]. Such sampling methodologies are portrayed as being created by a series of referrals that are made within a circle of people who know one another. This cyclical nature permits loops, in which a person named in a later wave in turn names someone from an earlier wave, and so on [2],[34].

Snowball sampling was conceptually designed as a sample recruitment method that offered a way to overcome many of the recruitment challenges associated with inviting difficult-to-reach communities to join health-care research studies. The experience that was gained over many years of use generated a body of knowledge about both the benefits and limitations of this approach [26].

One of the benefits of snowball sampling is its potential to shorten the time and diminish the cost required to assemble a participant group of sufficient size and diversity from the specific target group [26],[34],[35]. Another benefit of snowball sampling is when the study's participants who are sought, they are so well integrated with the mainstream community that it is difficult to identify individual group members, as is often the case of MSM. Community informants can help to identify which individuals with potentially eligible criteria are actually from the community of focus [26]. This is even more pertinent when the study's eligibility criteria involve characteristics that some people consider to be very private (e.g. men's engagement in sex with other men). In this sense, a main benefit of snowball sampling is the possibility to reach particular most-vulnerable subgroups through community key-informants, who otherwise could not be identified by the researchers and included in the research. Additionally, a particular advantage of snowball sampling is the inherent trust it encourages among potential participants, which can help to increase the likelihood that the identified person will agree to talk with the researcher [26],[34].

Nonetheless, probability sampling methods are considered to be the gold standard for recruiting participants who are most likely to be representative of the larger population from which they are drawn. Thus, the downside to snowball sampling is that it is a non-probability method [26],[34]. Snowball sampling lacks validity in representation because the composition of the sample is dependent upon the choice of seeds (initial recruits) and the size of recruitment chains (the number of recruits per participant) [35]. Although initial seeds in snowball sampling may be in theory randomly chosen, in practice this is difficult, if not impossible, to carry out. As a practical matter, initial seeds in snowball sampling tend to be chosen via convenience sampling [16]. Also, the sample composition is heavily influenced by the choice of initial seeds, and in practice, the method also tends to be biased towards favouring more cooperative individuals, as opposed to randomly chosen subjects and those that are part of larger personal networks [16],[26],[35]. So, in a study that uses a snowball recruitment strategy, any conclusion reached may be particularly biased; for example, the sample may include an over-representation of individuals with numerous social connections who share similar characteristics [16],[19],[26]. In a study conducted in Fortaleza, Brazil in 2002–2005, community-based behavioural surveys of MSM were conducted as part of a comprehensive Second Generation HIV Surveillance system [35]. In that period, MSM were sampled through different sampling strategies, including the snowball method as well as intercepting and interviewing members of the MSM population in venues where MSM met, which was previously mapped through formative research. Interviewing MSM and asking them to identify others to be recruited to the survey made this method the least costly survey method in the study. However, the snowball sample comprised significantly more MSM in the higher social classes than other methods samples, and consequently data collected through snowball sampling over-represented men with financial resources [35].

Additionally, chain referral methods like snowball sampling do carry the inherent risk of disclosure of personal information to others. The participants may be reluctant to contact other individuals whom they believe to have a certain characteristic, as that may represent a disclosure of information about themselves or information that has been obtained in a personal and private context., When contacted by the source, the named individuals are faced with the challenge of deciding whether to disclose information about their personal status to the outsider, in order to claim their eligibility for participation in the research study [26],[34].

In face of the drawbacks of chain-referral methods, mostly related to its dependence on the characteristics and dynamics of the network of participants, alternative methods have been put forward, as venue-based methods.

#### 2.1.2. Venue-based Sampling

Venue-based techniques seek to recruit respondents in places and at times where they would reasonably be expected to gather, and to collect data from them within that place [36],[37]. In this sense, the initial phase of identification of venues relies on the specific characteristic(s) being sought (e.g. venues where MSM gather), and, for a comprehensive sampling, researchers need to collect information on the target population's attendance habits, such as frequency of attendance at the venue, frequency of attendance at other venues, and time spent in the venue [37]. Overall, the venue-based method allows convenient access to the target population without having to rely on participants and network connections, therefore circumventing some of the biases associated with chain-referral methods.

Nevertheless, some sampling biases may also be introduced in using venue-based method. A significant limitation is the assumption that all members of the target population attend venues that researchers can access [36],[37]. Actually, some members of the population may not attend the venues or may do so very rarely, having a near zero probability of being recruited compared with population members who attend often. For example, in a research project aimed to evaluate the effects of multicomponent, community level intervention for promoting safer sex behaviour among young 15–25 year old MSM in United States of America, the venue-based method was used to recruit participants for the surveys, and some limitations were described [37]. Young MSM have characteristics which make them a hard-to-reach population, even with a venue-based approach [37]. Due to age restrictions for entering many establishments where MSM socialize, young men are less likely to be found at venues such as bars. In addition, young MSM from ethnic minority populations may identify more with their ethnic groups than with the gay community, and may not frequent well-known gay-identified establishments [37]. Also, the venues which researchers are able to visit and where they collect data may themselves introduce a bias. This is particularly pertinent in research aimed to provide estimates of HIV prevalence and risk behaviours, as different venues where MSM socialize present disparate levels of prevalence of HIV infection and high-risk sexual behaviours, as shown in the literature [38]–[40]. Another limitation is that individuals attending highly-frequented venues may have a lower probability of being enrolled than those attending low-density venues [37]. The information on the target population's frequency of attendance at the venue is sometimes difficult to assess and mainly depends on the accuracy of attendees' recall. Moreover, having a complete or representative list of venues and attendance times is a key step to ensure that the method is efficiently carried out; however, reliance on the identified universe of venues and times may lead to a bias of over- or under-representation of venues attended by some social networks [37].

#### 2.1.3. Combination of Methods

Given the limitations in reaching and collecting relevant data from particularly difficult-to-access subgroups of most-at-risk populations as MSM, several recent studies have been using combinations of snowball/chain referral sampling methods and venue-based recruitment strategies [36],[40]–[42]. As a non-probability sampling approach, the combination with venue-based sampling extends the ideas of snowball sampling to include a formative research consisting in an initial ethnographic assessment aimed at identifying the various networks or subgroups that might exist in a targeted setting [16],[43]. In such formative research, the adoption of community-based participatory research approach has been valued [44],[45]. This approach promotes community partners' involvement in the research process, and enables to integrate their expertise and knowledge on the study population' characteristics and contexts, allowing for a more accurate formative research [46]. The use of these two different approaches has contributed to obtaining broad samples of most vulnerable populations. In a participatory HIV behavioural survey conducted with MSM in Portugal, combining venue-based and chain referral sampling methods allowed investigators to reach a sample of 1,046 MSM comprised of diverse subgroups potentially difficult-to-access, as those unemployed and with lower incomes, reporting high-risk taking behaviours, HIV-positive and those never tested for HIV [38]. The study had a high acceptance from MSM, which translated into high recruitment and retention rates—overall, 76.8% of MSM accepted to participate in the survey [38]. This was possible due to the recruitment strategy adopted. Recruitment teams of outreach workers from local non-governmental organizations working on HIV prevention, and MSM peers from community-based organizations were sent to different venues where MSM gathered to recruit participants, previously mapped through a formative research developed by the Community Advisory Board of the project. Simultaneously, respondents were asked to advertise the study among their social networks, in an attempt to reach potential eligible peers [38]. Nevertheless, a main limitation of the study, that has also been reported in several other research using similar sampling methods, is the impossibility to infer the results from the study sample to the MSM in general due to the fact that the study sample was not randomly recruited [2],[16],[38].

As a major concern of non-probabilistic sampling methods being the fact that the selection bias limits the validity of the sample and potentially the quality of data, attempts have been made to improve the statistical accuracy of samples obtained by these techniques [34].

### 2.2. Respondent-driven Sampling

A sampling method specifically designed to overcome some of the biases associated with traditional chain-referral sampling methods is Respondent-driven sampling (RDS) [47]. RDS has rapidly become popular and widely used, being applied in more than 120 studies in over 20 countries involving more than 30,000 participants [48],[49]. RDS has being used for surveillance of populations most-at-risk for HIV/AIDS as MSM, injecting drug users and sex workers in many countries worldwide [35],[49],[50]. A systematic literature review found that MSM were more frequently recruited by semi-probabilistic methods as RDS, in line with the WHO recommendations on methods for conducting HIV surveillance among populations most at risk for HIV [33],[51]. Particularly, RDS has been currently used by the U.S. Centers for Disease Control and Prevention to help track the HIV epidemic and provide data for public-health decision-making [48],[52],[53].

RDS presents two key innovations for sampling difficult-to-reach populations as MSM: a design for sampling from the target population that relies on a structured system of recruitment procedures, involving recruitment of peers by their peers, a dual system of incentives and a coupon system; and a corresponding strategy for estimating properties of the target population based on the resulting sample, employing post-stratification weighting procedures [16],[43],[47],[48],[53],[54].

The recruitment starts with an initial set of seed respondents, usually selected by convenience sampling or other methods, who are given coupons to recruit others from the target population [16],[47],[52]. The recruitment process continues in waves, with seeds recruiting first-wave respondents, first-wave respondents recruiting the second-wave respondents, and so on until a pre-determined sample size is achieved or an equilibrium is reached – the point at which sample characteristics cease to fluctuate and theoretically approximate the characteristics of participants' networks [16],[47],[52],[54]. The coupon system is used to monitor the number of peers one can recruit into the study, and recruitment information is used to link recruiters to recruits [43],[47]. Respondents are typically given an incentive (often money) for interview completion, and then for each peer successfully recruited [16],[43],[47],[52],[54].

Afterwards, estimation methods are applied to account for the non-random sample selection in an attempt to generate unbiased estimates for the target population [43],[52]. Individuals with more contacts in the target population are more likely to be recruited [55]. Therefore, to adjust for this selection bias, respondents are asked to estimate how many people they know in the hidden population, and the inverse of each person's estimate is then used as a weight to discount the respondents most likely to be sampled [48],[53].

The RDS method is similar to snowball sampling in that it involves chain-referral sampling, but the implementation of the recruitment process allows for the calculation of selection probabilities [16]. In addition, the method has greater external validity because it is not limited to subgroup members who are accessible at sites, but rather extends the sample to all potential members of a subgroup selected for surveillance by accessing respondents through their social networks [16]. Several studies conclude that RDS is an appropriate, efficient and fast way to recruit large samples of hard-to-reach populations as MSM, highlighting that the demographics of samples recruited through RDS reflect the general demographics of the population in similar study locations [16],[47],[50],[52],[54],[56]. RDS lends statistical rigor to conventional snowball sampling through longer recruitment chains, recruitment limits, and the collection of data used to statistically adjust the biases inherent in how persons of similar characteristics are networked and likely to recruit each other [28],[35],[56],[57]. A main advantage of RDS over other non-probability methods for sampling hidden populations is that the long sampling chains reduce, or ideally eliminate, the biases induced by the convenience sampling of seeds [43].

However, as with all population-based studies, RDS is not immune from sampling bias as shown in several studies [47],[48],[52],[54]. Some of those biases include differential recruitment effectiveness (when some groups are better at recruiting than others), differential recruitment patterns (also known as homophily, the individuals' tendency to associate with other individuals with similar socio-demographic and behavioural characteristics), and heterogeneity in degree (differences between groups in terms of network size, with subjects in larger network being over-sampled because more recruitment paths lead to them) [47],[52]. Other studies have shown that recruitment efficiency of RDS may vary by population density and by the ability to recruit productive seeds [54]. Post-stratification weighting is proposed as a way to correct sampling bias in RDS samples, but it cannot fully account for bias introduced by non-random selection from personal networks [54]. Indeed, such weighting is a mathematically appropriate technique for characterizing recruitment probabilities in a sample from a network if RDS assumptions are met and respondents are able to estimate their popularity with reasonable accuracy, which is not always the case [53],[54].

These biases may increase the design effect of respondent-driven sampling, which measures the increased variation of the estimates [52],[58]. While RDS is an efficient means of recruiting samples of hidden populations, inaccuracies in estimates of network sizes may lead to biases [54]. Recent research indicates that the accuracy of the RDS approach is sensitive to the assumptions it makes about the social network of the underlying population [43],[48],[53]. Indeed, the accuracy of RDS estimates is affected by the structure of the underlying social network, the distribution of traits within the network, and the recruitment dynamics [48],[53]. Consequently, according to some studies assessing RDS efficiency, RDS estimates are much less precise and its variability is significantly larger than generally believed [48],[52]. Overall, RDS statistical-inference methods can fail and the confidence intervals may be too narrow [52]. A study investigating the performance of RDS by simulating sampling from 85 known network populations of sex workers and drug users found that variance of RDS is typically 5–10 times greater than that of simple random sampling and, moreover, standard RDS confidence intervals are misleadingly narrow [48]. In this sense, the authors put forward that some RDS studies may lack sufficient power to identify changes in behaviour and disease prevalence with statistical confidence [48]. This implies collecting larger sample sizes than would be needed from random sampling to maintain the same level of statistical power [53].

### 2.3. Time-location Sampling

Another approach that has seen increasing use in recent years is Time-location sampling (TLS). This is a venue-based method that came into use to sample hard-to-reach populations, primarily MSM, in the late 1980s to early 1990s [20],[59], and takes advantage of the fact that some hidden populations tend to gather or congregate at certain types of locations [16],[29],[37]. MSM often congregate in commercial and non-commercial venues, as gay bars and “cruising areas”, known to attract MSM [16],[38]. In TLS, such venues are enumerated in a preliminary ethnographic mapping or formative research exercise. The method entails identifying days and times when the target population congregates at the specific locations, constructing a sampling frame from which to choose a probability sample of time and location units [16],[20],[35],[37],[60]. Data are gathered from either all or a sample of subgroup members found at the site during the pre-defined time interval. The number of group members at each location provides a sampling weight that can be a priori used to draw a self-weighting sample, or post priori in analysis [16]. Because probabilities of selection can be calculated, TLS qualifies as a probability sampling method [16],[20],[29],[31],[37].

The major contribution of TLS over other cluster sampling methods is the ability to account for the fact that populations of interest are not statically associated with a particular location, and often move between multiple locations during the course of a single day. As such, TLS allows researchers to construct a sample with known properties, make statistical inference to the larger population of location visitors, and theorize about the introduction of biases that may limit generalization of results to the target population [20],[31]. TLS has been used widely for routine biological and behavioural surveillance surveys among most-at-risk populations that are concentrated in specific geographical areas that may “float” among locations [20],[61]–[63].

In an HIV bio-behavioural survey implemented in nine European cities and countries, a total sample of 3,661 MSM was reached, recruited through TLS in a diverse set of physical public or private locations, including commercial venues such as cafes, discos/clubs, bars, sex shop, sex cinema, saunas, spas, etc. as well as non-commercial venues, such as cruising settings and special events [64]. Excluded were venues that specifically serve HIV positive members of the priority population. Indeed, including these types of venues would introduce sampling biases by artificially increasing representation of HIV-positive individuals in the final sample [64]. The construction of sampling frames is allowed to generate a statistically representative sample from “hidden” and most-at-risk populations among MSM. Once the initial list of venues was elaborated based on the findings of the formative research, MSM venues and venue-day-time (VDT) units were identified and two sampling frames constructed. The first sampling frame (or venues sampling frame) comprised a list of venues that met the attendance requirements and were also willing to participate (eligible venues). The second sampling frame (VDT sampling frame) comprised a list of venue-specific sampling periods of four hours each. Following completion of the final sampling frame, a three-stage sampling plan to select venues, VDTs, and participants was used [64]. This semi-probabilistic method enabled to obtain relevant evidence for MSM prevention campaigns and for effective epidemiological surveillance, based on the estimation of HIV/STI prevalence and of the undiagnosed infections in the MSM population, the identification of sexual risk behaviour patterns, and a detailed knowledge of the heterogeneity of prevention needs in different contexts [65].

However, TLS method has several limitations. Some locations may be missed, particularly sites that are exceptionally discrete, while others may not have sufficient numbers of eligible group members [16],[20]. A significant proportion of subgroup members may tend not to frequent the selected sites, which is relevant given that the behaviours and HIV status of subgroup members who do not visit gathering venues differ from those who do [16],[20],[37]. Logistically, identification of all gathering venues can in theory be achieved given sufficient time and resources for sampling frame development, but there are practical limits to the resources that can be committed to such activities on a regular basis [16]. Also, the venues where members of MSM subgroups congregate frequently change over time resulting from economical or law enforcement actions, which implies updating the sampling frame before each round of data collection, and this has high costs associated with it [16],[20]. The nature of the recruitment sites is itself a limitation. MSM attending bars and dance clubs may not want to participate in biobehavioural surveys in which they might learn their HIV status [16]. Some recruitment venues may also be unsafe, with the potential to jeopardize community researchers' security [20]. Finally, accounting for non-participation may be challenging when owners forbid recruitment on their property, or in public locations where it is impractical or illegal to recruit participants [20]. A major question is whether non-response is differential in diverse settings when subgroup members are approached to participate in a surveillance survey [20]. All these limitations of TLS method are potentially important sources of sampling bias.

### 2.4. Internet Sampling

With increasing access to the Internet and growing popularity among MSM in seeking sex partners online, the Internet has also been increasingly used to recruit MSM in HIV research [66],[67]. The advantages of using this method are faster recruitment, lower operational costs and a greater level of anonymity provided to participants, which allows potentially capture more MSM without self-disclosure of their sexual orientation, compared with those recruited in person and from venues [36],[68]–[70]. Also, Internet sampling is the method that reaches a higher number of respondents. A systematic literature review showed that Internet sampling reached a total of 225,320 participants in 33 selected studies, compared to 82,004 participants reached by RDS in 77 studies, and 55,193 participants reached by TLS in 42 studies [51]. One of the strengths of the European MSM Internet Survey (EMIS), administered across 38 European countries, was that it reached the largest and most geographically diverse dataset to examine country-level stigma and the pathways through which it operates to suppress both HIV-risks and HIV-precautions among MSM [67].

Nevertheless, Internet sampling is subject to selection biases, as it can only sample those MSM who have access to the Internet, and may sample particular subgroups who engage in certain risk behaviours associated to Internet use. For instance, in EMIS the participants who were reached differed from the broader population of MSM, over-representing younger men and men with diagnosed HIV. Additionally, the degree and direction in which this selection bias may under- or overestimate the relationship between sample characteristics and HIV outcomes is difficult to predict and control [67],[69].

The advantages and limitations of each sampling strategy are summarized in Table 1.

**Table 1.** Advantages and limitations of each sampling strategy.

Sampling strategy	Advantages	Limitations
Snowball sampling	Short time and reduced cost to assemble a large and diverse participant group. Trust of potential participants and increased likelihood that they will agree to talk with the researcher. Collaboration of community informants to identify and reach most-vulnerable subgroups from the community under study.	Non-probability method lacks validity in representation. Sample composition heavily influenced by the choice of initial seeds. Potential bias towards favouring more cooperative, as opposed to randomly chosen subjects, and those that are part of larger personal networks. Inherent risk of disclosure of personal information to others, participants may be reluctant to disclose information about themselves or information about their peers that has been obtained in a personal and private context.
Venue-based sampling	Convenient access to the target population without having to rely on participants and network connections, while also avoiding some biases associated with chain-referral methods.	Lack of access to some venues attended by the target population. Potential to not reach some members of the population as they may not attend the identified venues. Difficulty to access accurate information about the target population's frequency of attendance at the venue. Potential bias of over/under-representation of venues attended by some social networks due to reliance on the identified universe of venues and times.
Respondent-driven sampling	Estimation methods applied in an attempt to generate unbiased estimates for the target population. Greater external validity. Lends statistical rigor to snowball sampling through: longer recruitment chains recruitment limits collection of data used to statistically adjust for the recruitment biases.	Differential recruitment effectiveness (when some groups are better at recruiting than others) Differential recruitment patterns (homophily: individuals' tendency to associate with other individuals with similar characteristics; heterogeneity in degree: differences between groups in terms of network size, with subjects with larger network sizes being over-sampled). Implies collecting large sample sizes to ensure statistical power.
Time-location sampling	Construct a sample with known properties, allows theorize about the introduction of biases that may limit generalization of results to the target population. Possibility to make statistical inference to the larger population of location visitors.	Some locations may be missed, particularly sites that are exceptionally discrete, while others may not have sufficient numbers of eligible group members. The venues where members of MSM subgroups congregate frequently change over time. The nature of the recruitment sites itself may reduce the acceptability of potential participants to enrol in the study and/or can affect the disclosure of information by participants.
Internet sampling	Faster recruitment. Lower operational cost. Greater level of anonymity provided to participants. Reaches higher number of respondents.	Selection bias, it can only sample those who have access to the Internet. Potential over-sampling of subjects with higher levels of internet use and/or users of higher number of online networks.

## 3. Conclusions

Given the critical importance of understanding HIV epidemics, high quality surveillance systems are necessary. The need to find more effective and efficient recruitment strategies is paramount. Appropriate sampling approaches are at the core of any high quality surveillance system, especially when attempting to track transmission dynamics among populations that play a critical role in the transmission of HIV, and that are “hidden” or hard-to-reach [16],[26]. The varying approaches face common challenges. The definition of the study population continues to be challenging as there is no consensus on who the MSM group includes (consider men who had only anal sex or also oral sex? Men had sex with men in their lifetime or in the last year?). According to UNAIDS Terminology Guidelines, the term men who have sex with men “describes males who have sex with males, regardless of whether or not they also have sex with women or have a personal or social gay or bisexual identity” [71]. This is a useful concept because it also includes men who self-identify as heterosexual but who occasionally have sex with other men. Indeed, an additional global challenge has been to identify this especially hard-to-reach MSM subgroup. Nevertheless, the methods discussed in this paper represent some of the best efforts thus far to find feasible sampling approaches and recruitment strategies that effectively reach most-at-risk and hard-to-reach subgroups of MSM, contributing to obtain unbiased trends of HIV prevalence and HIV-related risk behaviours among these populations.

The benefits and limitations of the described strategies should be carefully evaluated against its benefits and limitations in each specific context, in order to select the optimal strategy. Also, the benefits of using these approaches to recruit participants for HIV research must be weighed against the privacy concerns that are inherent in any individual social situation or health condition [26].

In recent years, the important role of community-based participatory approach in undertaking research with hard-to-reach populations has increasingly been recognized [38],[44],[45]. Participatory research integrates a collaborative approach with involvement of communities, professionals, political decision-makers, and academics to produce knowledge, incorporating the different perspectives and experiences of these stakeholders [46]. Community-based participatory approach may particularly help researchers choose the methods that most appropriately target MSM, relying on community partners and “insiders” knowledge on the best strategies to locate and approach these groups.
